# Trends in Atrial Fibrillation‐Related Mortality Among Adults With Obesity in the United States From 1999 to 2024

**DOI:** 10.1002/osp4.70160

**Published:** 2026-06-10

**Authors:** Aymen Ahmed, Ruqiat Masooma Batool, Saad Ahmed Waqas, Hasan Fareed Siddiqui, Muhammad Sameer Arshad, Neha Saleem Paryani, Izza Shahid, Hasibullah Aminpoor, Ali Mithani, Muhammad Talha Ayub

**Affiliations:** ^1^ Endeavor Center for Cardiovascular Intervention Outcomes Research and Evaluation (ECCORE) Section of Interventional Cardiology Endeavor Health Cardiovascular Institute Glenview Illinois USA; ^2^ The University of Chicago Pritzker School of Medicine Chicago Illinois USA; ^3^ Department of Medicine Dow University of Health Sciences Karachi Pakistan; ^4^ Baylor Scott and White Research Institute Dallas Texas USA; ^5^ Department of Medicine HCA Houston Healthcare Clear Lake/University of Houston Webster Texas USA; ^6^ Division of Preventive Cardiology Houston Methodist Academic Institute Houston Texas USA; ^7^ Department of Internal Medicine Rabia Balkhi National Complex Hospital Kabul Afghanistan; ^8^ Faculty of Medicine Kabul University of Medical Sciences “Abu Ali Ibn Sina” Kabul Afghanistan; ^9^ Department of Cardiac Electrophysiology Baylor Scott & White The Heart Hospital Plano Texas USA; ^10^ Division of Cardiac Electrophysiology ProMedica Physicians Cardiology Toledo Ohio USA

**Keywords:** atrial fibrillation, CDC WONDER database, epidemiological trends, health disparities, mortality, obesity

## Abstract

**Background:**

Atrial fibrillation (AF) is a prevalent cardiac arrhythmia associated with increased mortality, particularly among individuals with obesity. This study examined trends in AF‐related mortality in adults with obesity in the United States from 1999 to 2024.

**Methods:**

Trends in AF‐related mortality in adults with obesity aged ≥ 25 years with obesity were analyzed using the Centers for Disease Control and Prevention Wide‐ranging Online Data for Epidemiologic Research (CDC WONDER) database. AF and obesity were considered as multiple causes of death. Age‐adjusted mortality rates (AAMRs) per 1,000,000 were analyzed using Joinpoint regression to identify trends and calculate average annual percentage change (AAPC) stratified by gender, race, and region.

**Results:**

A total of 63,853 deaths were identified. From 1999 to 2024, AF‐related mortality rates increased from 1.9 in 1999 to 20.8 in 2024 (AAPC: 11.4 [95% CI: 10.4, 12.2]). Males recorded higher AAMRs than females. White individuals exhibited higher AAMRs than Blacks. Nonmetropolitan areas (9.6) had higher AAMR than metropolitan areas (7.4). The Midwest recorded the highest AAMR while the South reported the steepest rise. States in the top 90th percentile of AAMR were Vermont, Oklahoma, Oregon, Minnesota, and Wyoming.

**Conclusion:**

AF‐ and obesity‐related mortality has increased overall from 1999 to 2024, with higher mortality rates observed in men, White individuals, nonmetropolitan areas, and the Southern US region. These findings highlight the need for continued surveillance and targeted prevention efforts in this population.

## Graphical abstract

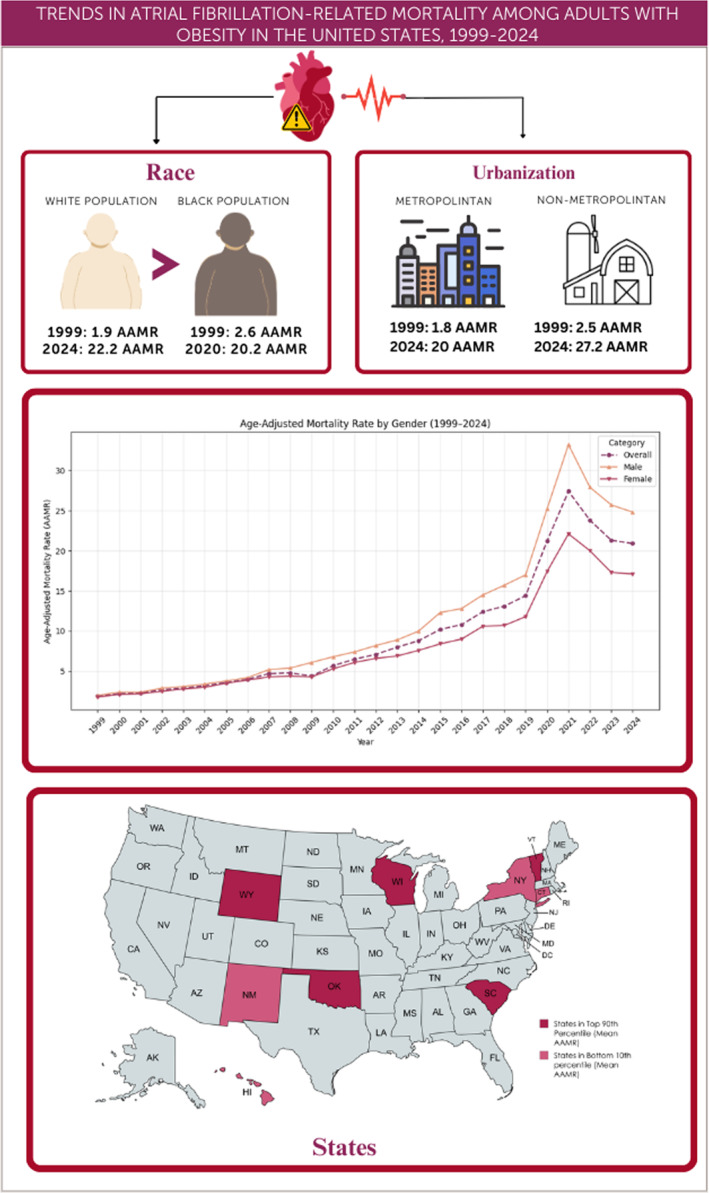

**Abbreviations**
AAMR: age‐adjusted mortality rate
AAPC: average annual percentage change
AF: atrial fibrillation
APC: annual percent change
BMI: body mass index
CDC: Centers for Disease Control and Prevention
CI: confidence interval
DOACs: direct oral anticoagulants
EAT: epicardial adipose tissue
ECG: electrocardiogram
ICD‐10: International Classification of Diseases, 10th revision
IRB: Institutional Review Board
MRI: magnetic resonance imaging
NCHS: National Center for Health Statistics
RCT: randomized controlled trial
SNP: single nucleotide polymorphism
STROBE: strengthening the reporting of observational studies in epidemiology
US: United States
WONDER: wide‐ranging online data for epidemiologic research

## 1 Introduction

Atrial fibrillation (AF) is the most prevalent cardiac arrhythmia that is increasingly recognized as a primary contributor to cardiovascular morbidity and mortality [1, 2]. The global burden of AF has risen dramatically, with cases climbing from 33.5 million in 2010 to nearly 59 million in 2019. This upward trend is expected to continue, with projections estimating a further increase of more than 60% by 2050 [3, 4]. Parallel to this, obesity, declared a global epidemic by the World Health Organization [5], has become a severe public health challenge, now affecting 35% of US adults aged 65 and older and contributing to increased mortality rates [6]. Severe obesity prevalence is projected to rise by 130% over the next 2 decades, with the number of older adults with obesity anticipated to reach 82.3 million by 2040 [1, 7].

The convergence of AF and obesity in older adults represents a growing public health concern and imposes a significant economic burden on healthcare systems. The association between AF and obesity is well documented. Evidence from the Framingham Heart Study showed that obesity raises the risk of new‐onset AF by 50%, and a 5‐unit increase in body mass index (BMI) correlates with a 30% rise in AF incidence [8]. Several shared pathophysiological mechanisms underlie this link, including chronic inflammation, myocardial fibrosis, adipocyte infiltration of cardiac tissue, and the clustering of cardiometabolic comorbidities, all of which increase susceptibility to AF [9]. Although AF is rarely a direct cause of death, “AF‐related mortality” in this study refers to deaths where AF is recorded as an underlying or contributing cause, often reflecting downstream complications such as cardioembolic stroke, heart failure exacerbation, systemic thromboembolism, or arrhythmia‐related sudden cardiac death.

Despite alarming trends in AF‐related mortality, the impact of obesity on AF prognosis remains complex and underexplored. Some studies indicate that weight reduction may improve AF outcomes, yet others report a favorable prognosis for AF among patients with obesity, a phenomenon termed the “obesity paradox [10, 11]. To fill existing knowledge gaps, this study investigates AF‐related mortality trends among individuals with obesity from 1999 to 2024. Drawing on data from the CDC's Wide‐Ranging Online Data for Epidemiologic Research (WONDER) system, this analysis examines variations across sex, race, urbanization, and geographic regions to identify vulnerable populations and provide a detailed understanding of these intersecting health concerns.

## 2 Methods

### 2.1 Study Setting and Population

A descriptive analysis was conducted utilizing death certificate data sourced from the Centers for Disease Control and Prevention's WONDER (Wide‐Ranging Online Data for Epidemiologic Research) database. Our primary objective was to assess the mortality rates related to obesity and AF among individuals aged ≥ 25 years from 1999 to 2024. This analysis focused on death certificates included in the Multiple Causes of Death Public Use dataset. The International Statistical Classification of Diseases and Related Health Problems, 10th Revision (ICD‐10) codes E66 (obesity) and I48 (AF) were used to identify deaths where these conditions were recorded as the underlying or contributing cause. The term AF‐related mortality refers to deaths in which AF was listed as either the underlying cause or a contributing cause on the death certificate. We did not require Institutional Review Board approval, as we used a de‐identified, government‐provided public‐use dataset, following the Strengthening the Reporting of Observational Studies in Epidemiology (STROBE) guidelines.

### 2.2 Data Abstraction

The data were categorized by demographic factors, including age, gender, race/ethnicity, urbanization status, and state of residence. Racial/ethnic groups were defined as White American and Black or African American. The study population was geographically categorized using the Urban‐Rural Classification Scheme from the National Center for Health Statistics, with urban areas including small to large metropolitan regions and rural areas encompassing locales with populations of less than 50,000 individuals [12]. The US was divided into four regions based on the US Census Bureau's classification: Northeast, Midwest, South, and West. Urbanization data were not available from 2020 onward.

### 2.3 Statistical Analysis

Obesity and AF‐related mortality patterns were analyzed from 1999 to 2024, considering gender, race, age, urbanization status, and state of residence. Crude and age‐adjusted mortality rates (AAMR) per 1,000,000 individuals were calculated, using the 2000 US population as a baseline for AAMR standardization. To evaluate temporal changes in mortality rates, the Joinpoint Regression Program (Version 5.0.2, National Cancer Institute) was utilized. This approach involved fitting log‐linear regression models to the data trends to calculate the annual percent change (APC) in AAMR and 95% confidence interval (CI). APCs were categorized as increasing or decreasing based on their statistical deviation from the null hypothesis of zero change, with statistical significance determined by using a two‐tailed *t*‐test and a threshold of *p* < 0.05.

## 3 Results

A total of 63,853 AF‐ and obesity‐related deaths occurred among adults (aged ≥ 25 years) between 1999 and 2024. Information on the location of death was available for 61,875 deaths. Among these, 52.5% occurred in medical facilities, 31.9% at home, 12.5% in nursing homes/long‐term care facilities, and 3.0% in hospices.

### 3.1 Annual Trends for AF and Obesity‐Related AAMR

**FIGURE 1:**
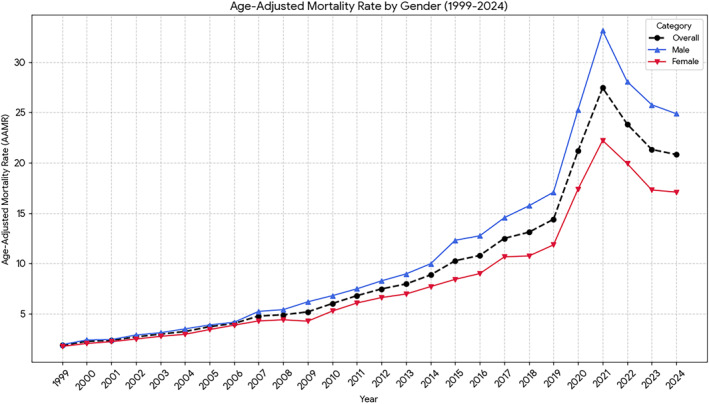
Obesity and atrial fibrillation age‐adjusted mortality rates per 100,000, stratified by sex in the United States, 1999 to 2024.

The AAMR for AF and obesity‐related deaths in adults was 1.9 in 1999 and 21.2 in 2020 (average annual percentage change [AAPC]: +11.4%; 95% CI: 10.4–12.2). The overall AAMR increased from 1999 to 2018 (APC: +10.2%; 95% CI: 9.3–11.1), followed by a sharp rise from 2018 to 2021 (APC: +26.5%; 95% CI: 21.0–29.7), and subsequently declined from 2021 to 2024 (APC: −8.6%; 95% CI: −12.2 to −5.5) (Figure 1, Supporting Information S1: Table S1). Upon sensitivity analysis, AAMR for AF alone and obesity alone as underlying or contributing causes of death showed increasing trends (Figure S1).

### 3.2 AF and Obesity‐Related AAMR Stratified by Sex

Among females, the AAMR rose steadily between 1999 and 2018 (APC: +9.5%; 95% CI: 8.5–10.3), followed by a marked increase from 2018 to 2021 (APC: +25.8%; 95% CI: 19.9–29.2). Thereafter, rates declined from 2021 to 2024 (APC: −8.4%; 95% CI: −12.1 to −5.2). In males, mortality trends showed a similar pattern, with an increase from 1999 to 2018 (APC: +11.1%; 95% CI: 10.0–12.0), accelerating between 2018 and 2021 (APC: +26.9%; 95% CI: 20.8–30.3), before decreasing from 2021 to 2024 (APC: −8.6%; 95% CI: −12.5 to −5.4) (Figure 1, Supporting Information S1: Table S1).

### 3.3 AF and Obesity‐Related AAMR Stratified by Race/Ethnicity

**FIGURE 2:**
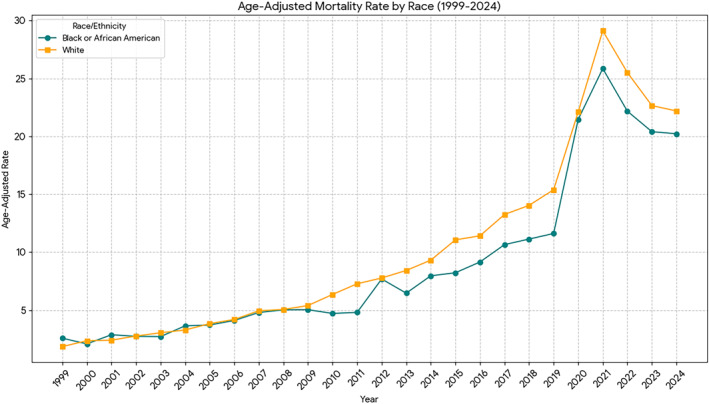
Obesity and atrial fibrillation age‐adjusted mortality rates per 100,000, stratified by race in the United States from 1999 to 2024.

Among Black or African American individuals, AAMR increased from 1999 to 2018 (APC: +8.6%; 95% CI: 7.1–9.7), followed by a pronounced surge between 2018 and 2021 (APC: +33.6%; 95% CI: 23.1–39.6). This was subsequently followed by a decline from 2021 to 2024 (APC: −9.0%; 95% CI: −14.2 to −4.3). In White individuals, mortality rates rose between 1999 and 2018 (APC: +10.5%; 95% CI: 9.6–11.4), with a further increase during 2018–2021 (APC: +26.0%; 95% CI: 20.5–29.2), before decreasing from 2021 to 2024 (APC: −8.3%; 95% CI: −11.9 to −5.2) (Figure 2, Supporting Information S1: Table S2).

### 3.4 AF and Obesity‐Related AAMR Stratified by Geographic Region

**FIGURE 3:**
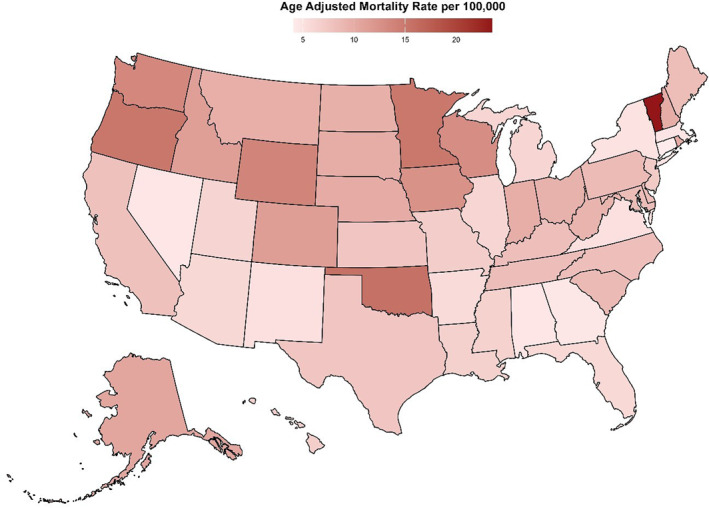
Obesity and atrial fibrillation age‐adjusted mortality rates per 100,000, stratified by state in the United States from 1999 to 2020.

A significant difference in AAMRs was observed in different states, with the AAMRs ranging from 4.1 (95% CI: 3.5–4.6) in Connecticut to 23.5 (95% CI: 20.6–26.4) in Vermont. States that fell into the top 90th percentile were Vermont, Oklahoma, Oregon, Minnesota, and Wyoming, which had around triple the AAMRs compared with states that fell into the lower 10th percentile, namely, Massachusetts, Nevada, Alabama, Georgia, and Connecticut (Figure 3, Supporting Information S1: Table S3). Overall state‐level data for 2021–2024 are presented in Figure S2 and Supporting Information S1: Table S4.

Across census regions, AAMR increased from 1999 to 2018 in the Northeast (APC: +9.5%; 95% CI: 7.1–10.8), Midwest (+10.7%; 95% CI: 9.6–11.6), South (+10.3%; 95% CI: 9.5–11.2), and West (+10.1%; 95% CI: 8.4–11.3). A sharper rise occurred between 2018 and 2021 in all regions—Northeast (+21.5%; 95% CI: 12.4–26.3), Midwest (+23.9%; 95% CI: 17.7–27.5), South (+32.2%; 95% CI: 27.2–35.5), and West (+24.0%; 95% CI: 15.1 to 27.9)—followed by declining trends from 2021 to 2024 (Northeast −8.5%; 95% CI: −15.4 to −3.4; Midwest −9.8%; 95% CI: −13.3 to −6.4; South −7.1%; 95% CI: −10.2 to −4.2; West −9.8%; 95% CI: −14.9 to −5.8) (Figure S3, Supporting Information S1: Table S5).

Nonmetropolitan areas had higher AF and obesity‐related AAMRs than metropolitan areas, with overall AAMRs of 9.6 (95% CI: 9.4–9.9) and 7.4 (95% CI: 7.3–7.4), respectively. AAMRs of nonmetropolitan areas showed a greater increase compared to metropolitan areas from 1999 to 2020 (nonmetropolitan: AAPC: +12.0%; 95% CI: 11.5 to 12.7; metropolitan: AAPC: +11.3%; 95% CI: 10.1–12.4) (Figure S4, Supporting Information S1: Table S6).

### 3.5 Top Underlying Causes of Death

Among patients with concomitant AF and obesity, heart diseases contributed the most to mortality as the underlying cause of death (25,105 deaths), followed by diabetes mellitus (4998 deaths) and chronic lower respiratory diseases (4478 deaths).

## 4 Discussion

This study examining AF‐related mortality trends among individuals with obesity between 1999 and 2024, revealed several key findings. First, a persistent upward trend in mortality from 1999 to 2018 was observed, followed by a marked acceleration through 2021 and a gradual decline till 2024. Second, mortality rates were significantly higher among White individuals relative to Black individuals. Third, males exhibited slightly higher rates of mortality than females. Fourth, non‐metropolitan areas were found to have marginally higher mortality rates than metropolitan areas, and the Southern US region exhibited the steepest increase in mortality rates overall. Moreover, states in the top 90th percentile for AAMRs experienced mortality rates approximately 3 times greater than the states within the bottom 10th percentile.

This is the first study that comprehensively analyzed CDC WONDER mortality data to identify national mortality trends specifically among individuals with obesity with AF. This association is supported by extensive prior literature. A study by *Lee* *et al.* on “metabolically healthy individuals with obesity” (BMI ≥ 30 kg/m^2^) established that obesity independently elevates AF risk, with a potential increase of up to 20% [13, 14]. On the contrary, some studies have reported paradoxical reductions in overall mortality and cardiac death among AF patients with obesity. While previous studies have either emphasized the AF‐obesity link or identified mortality trends, this study adds to the literature by revealing a rising national trend in AF‐related mortality among individuals with obesity.

Throughout the study period, a rising mortality rate was observed from 1999 to 2021, followed by a modest decline through 2024. This trend may be partly explained by demographic aging, with the older adult population projected to increase from 40.2 million to 88.5 million by 2050, accompanied by a higher prevalence of AF and obesity [15, 16]. Obesity frequently coexists with cardiovascular comorbidities such as hypertension, diabetes, and dyslipidemia, which exacerbate AF severity and contribute to increased mortality [17]. Additionally, age‐related functional changes such as endothelial dysfunction and vascular stiffening increase the risk of AF and subsequently, mortality [18]. Advances in imaging modalities, particularly cardiac CT and MRI, have improved the detection of visceral adipose tissue, strengthening evidence of its association with AF [19]. Obesity further complicates AF management by altering the pharmacokinetics of anticoagulants and antiarrhythmic drugs and is associated with [20] higher AF recurrence following ablation, thereby reducing effectiveness of rhythm control therapies [21, 22]. The sharp increase in mortality after 2019 may partly reflect the impact of the COVID‐19 pandemic, as AF in infected patients is associated with more severe clinical outcomes [23, 24]. While the decline after 2021 may reflect healthcare stabilization, mortality displacement among high‐risk individuals, or potential misclassification of deaths, the terminal mortality rates in 2024 remain significantly elevated compared with pre‐pandemic levels. Collectively, these factors contribute to a rising burden of mortality that challenges the well‐defined obesity paradox—the idea that a higher BMI may offer a protective effect in AF—suggesting instead that the long‐term risks of obesity‐mediated AF outweigh any proposed benefits.

This study focused on AF‐related mortality, and it is important to recognize that AF is rarely a direct cause of death. Instead, its presence on death certificates usually reflects its contribution to a more complex clinical course influenced by underlying comorbidities and related complications. In addition, mortality is rarely a primary outcome in randomized trials of AF, as the condition is not considered a malignant arrhythmia. For these reasons, mortality data attributed to AF should be interpreted with caution.

Males exhibited slightly higher AAMRs than females. However, the relationship between BMI and mortality in individuals with obesity and AF remains inconsistent across sexes. Data from the EORP‐AF Registry indicated that elevated BMI was associated with increased all‐cause mortality due to AF in female patients [25], whereas other studies report an inverse association (obesity paradox), where higher BMI is linked to lower mortality in women with obesity [25]. Overweight prevalence is higher in men (34.1%) compared with women (27.5%), which may account for the higher mortality rates in men [26]. Interestingly, female AF patients often present with more severe symptoms and reduced quality of life, potentially leading to earlier diagnosis and treatment [27, 28]. Biological differences may also contribute; estrogen‐related metabolic effects may improve myocardial energy utilization, while women tend to have greater subcutaneous fat deposition, which is metabolically protective compared with visceral adiposity more common in men [29, 30]. Further research is essential to clarify the sex‐specific mechanisms linking obesity and AF outcomes. White individuals experience higher AF‐related mortality rates than Black individuals, reflecting the “AF race paradox,” which highlights a greater AF prevalence in White individuals despite a higher prevalence of traditional risk factors such as obesity, hypertension, and diabetes among black individuals [31]. Paradoxically, obesity, which is a key risk factor for AF, is more prevalent among Black individuals (46.8%) than White individuals (37.9%) in older adults [32]. This disparity in mortality may be driven by genetic, physiological, and systemic factors. Black individuals are more likely to carry a protective PITX2‐related minor allele associated with lower AF risk, and greater European ancestry has been linked to higher AF incidence [33, 34]. Additionally, White individuals have greater visceral adiposity, which is strongly associated with AF, whereas Black individuals typically exhibit higher subcutaneous fat levels at similar BMIs [35].

Conversely, social and healthcare disparities likely contribute to under recognition of AF in Black populations. Limited access to care, lower health literacy, and underreporting may reduce AF detection and documentation in underrepresented groups [36, 37]. Atrial fibrillation is often intermittent and asymptomatic, and standard detection approaches that rely on self‐report, hospital records, or routine ECGs may fail to capture its true prevalence [38] Black individuals monitored with implanted cardiac devices are less likely to have AF detected compared to White individuals [39]. Moreover, biases in self‐reported height and weight, particularly among Black women, may distort BMI classification [40]. Together, these factors contribute to the apparent differences in mortality rates between racial groups.

AF‐related mortality among individuals with obesity is disproportionately high in the Southern and rural regions. Obesity prevalence in Southern areas often exceeds 40% and is accompanied by some of the highest rates of AF in the country as well [41, 42]. Rural areas, in particular, face compounded risks, with higher prevalence of smoking, hypertension, diabetes, and obesity, which are all critical risk factors for AF [43]. Older adults in rural regions face compounded healthcare challenges, including limited access to medical services, lower health literacy, reduced social support, and financial barriers, all of which hinder disease management and early detection. They also experience fewer screening opportunities, limited physician availability, and higher medication costs, exacerbating the risk of adverse outcomes [44, 45, 46].

Addressing AF in patients with obesity requires a comprehensive and multifaceted approach to improve outcomes and reduce mortality. Weight loss remains a pivotal intervention, with evidence demonstrating that reductions of more than 10% of body weight have been shown to meaningfully lower AF burden [47]. Additionally, improved cardiorespiratory fitness, whether through weight loss or independent training, further enhances prognosis [48]. Bariatric surgery offers additional benefits by improving insulin sensitivity, reversing adverse cardiac remodeling, and reducing epicardial adipose tissue, which is associated with fewer AF recurrences. Adopting a Mediterranean‐style diet may amplify these effects by further decreasing epicardial fat [49, 50]. Alongside lifestyle approaches, new treatment strategies are being developed to address the biological mechanisms linking epicardial adipose tissue to atrial fibrillation. These approaches focus on reducing inflammation and fibrosis through therapies that target adipokines, cytokines, growth factors, and oxidative stress [9]. Established AF management remains fundamental and includes rate control, rhythm control with antiarrhythmic drugs or catheter ablation, and stroke prevention with anticoagulation [5, 51].

To effectively manage AF in patients with obesity, further research is imperative to understand the underlying mechanisms. This includes the role of adipocyte phenotype heterogeneity, particularly the balance and distribution of white and brown adipose tissue, and how these differences may contribute to the development of arrhythmias [51]. Optimizing the dosing of direct oral anticoagulants (DOACs) in patients with morbid obesity remains a crucial challenge [51]. Further studies are needed to definitively establish optimal dosing regimens for this patient population. Additionally, measuring epicardial adipose tissue before a procedure may help predict the likelihood of atrial fibrillation recurrence [52]. The development of therapies specifically targeting epicardial fat has the potential to reduce inflammation and limit adverse structural remodeling, offering a promising direction for future treatment. Early risk stratification and personalized care are key to improving outcomes in obesity‐mediated AF [52]. It is also essential to prioritize the active inclusion and standardized reporting of underrepresented racial and ethnic groups in clinical trials to strengthen the evidence [53]. Lastly, a deeper understanding of the complex interplay between metabolic and inflammatory pathways, which link obesity to AF, may drive the development of novel therapies and ultimately improve patient outcomes [52].

This study has several notable limitations. First, reliance on ICD‐10 codes from death certificates may result in misclassification of both AF and obesity, and obesity is frequently underreported on death certificates, potentially underestimating its true contribution to mortality; furthermore, obesity‐related ICD codes have low sensitivity and may fail to capture many individuals with obesity in administrative datasets [54, 55]. Second, the E66 code cannot distinguish between obesity classes (Class I‐III) or assess body fat distribution patterns (visceral vs. subcutaneous), which may have distinct pathophysiological effects on AF risk. Third, the I48 codes do not differentiate between paroxysmal, persistent, or permanent AF subtypes, which may differ in their associations with mortality. Fourth, the dataset lacks clinical details such as BMI trajectories, duration of obesity/AF, as well as socioeconomic factors that may influence outcomes or treatment histories (e.g., anticoagulation, ablation). In addition, the lack of granular clinical data, including cardiovascular risk factors, comorbidities, and disease severity, as well as socioeconomic variables such as income, education, and insurance status, limits the understanding of disparities in care and outcomes. Another limitation is that the analysis focused only on Black and White populations due to insufficient mortality data for other racial and ethnic subgroups, which may limit the generalizability of the findings to the broader U.S. population. Fifth, advancements in medical treatments during the 1999–2024 period are not reflected in the analysis, which may influence the reported trends. Lastly, the cross‐sectional aggregate nature of the data prevents assessment of individual‐level and longitudinal relationships between AF, obesity, and mortality.

## 5 Conclusion

This study highlights the rising overall trend in AF‐ and obesity‐related mortality in the US from 1999 to 2024. Mortality rates were higher among men than women, with the highest rates observed in White individuals. Nonmetropolitan areas and the Southern US region exhibited notably elevated mortality rates over time. These findings emphasize the need for targeted interventions, including optimizing anticoagulation therapy in patients with obesity and leveraging biomarkers such as epicardial adipose tissue to enhance risk stratification. Additionally, prioritizing equitable access to care and addressing geographic disparities are crucial to mitigating the growing burden of mortality.

## Author Contributions

**Aymen Ahmed:** conceptualization, methodology, data curation, formal analysis, investigation, writing – original draft. **Ruqiat Masooma Batool:** conceptualization, data curation, investigation, writing – original draft. **Saad Ahmed Waqas:** conceptualization, methodology, data curation, formal analysis, software, investigation, visualization, writing – original draft. **Hasan Fareed Siddiqui:** methodology, data curation, formal analysis, software/statistical analysis, investigation, visualization, writing – original draft. **Muhammad Sameer Arshad:** methodology, formal analysis, software, visualization, writing – original draft. **Neha Saleem Paryani:** validation, writing – review and editing, resources. **Izza Shahid:** validation, writing – review and editing. **Hasibullah Aminpoor:** validation, resources, supervision, writing – review and editing. **Ali Mithani:** methodology, validation, resources, supervision, writing – review and editing. **Muhammad Talha Ayub:** methodology, validation, supervision, writing – review and editing.

## Funding

The authors have nothing to report.

## Ethics Statement

The authors have nothing to report.

## Consent

The authors have nothing to report.

## Conflicts of Interest

The authors declare no conflicts of interest.

## Permission to Reproduce Material From Other Sources

The authors have nothing to report.

## Supporting information


Supporting Information S1

**Figure S1:** Age‐adjusted mortality rates per 1,000,000 for obesity and atrial fibrillation as underlying or contributing causes of death, stratified by state in the United States, 1999–2020.

**Figure S2:** Obesity and atrial fibrillation age‐adjusted mortality rates per 1,000,000, stratified by state in the United States, 2021 to 2024.

**Figure S3:** Obesity and atrial fibrillation age‐adjusted mortality rates per 1,000,000, stratified by census region in the United States, 1999 to 2024.

**Figure S4:** Obesity and atrial fibrillation age‐adjusted mortality rates per 1,000,000, stratified by urbanization in the United States, 1999 to 2020.

## Data Availability

The data that support the findings of this study are openly available in CDC WONDER at https://wonder.cdc.gov/.
