# Supplementary material for: Trends in Atrial Fibrillation‐Related Mortality Among Adults With Obesity in the United States From 1999 to 2024
Source: Obes Sci Pract. 2026 Jun 10;12(3):e70160. doi: 10.1002/osp4.70160 (PMC13250834; doi:10.1002/osp4.70160)
Supplement: Supplementary file 1 — Supporting Information S1 [file OSP4-12-e70160-s002.docx]

**Supplementary Table 1.** Obesity and atrial fibrillation age-adjusted mortality rates per 1,000,000, stratified by sex in the United States, 1999 to 2024.

| **Year** | **Overall AAMR (95% CI)** | **Female AAMR (95% CI)** | **Male AAMR (95% CI)** |
| --- | --- | --- | --- |
| **1999** | 1.88 (1.68–2.08) | 1.77 (1.51–2.03) | 1.95 (1.64–2.27) |
| **2000** | 2.27 (2.05–2.49) | 2.06 (1.78–2.34) | 2.41 (2.07–2.75) |
| **2001** | 2.37 (2.15–2.60) | 2.24 (1.95–2.53) | 2.45 (2.11–2.78) |
| **2002** | 2.71 (2.48–2.95) | 2.50 (2.20–2.81) | 2.90 (2.53–3.27) |
| **2003** | 2.99 (2.74–3.24) | 2.77 (2.45–3.09) | 3.13 (2.76–3.51) |
| **2004** | 3.25 (3.00–3.51) | 2.97 (2.65–3.30) | 3.51 (3.11–3.91) |
| **2005** | 3.73 (3.46–4.00) | 3.44 (3.10–3.79) | 3.90 (3.49–4.31) |
| **2006** | 4.05 (3.77–4.33) | 3.88 (3.51–4.25) | 4.17 (3.75–4.59) |
| **2007** | 4.79 (4.49–5.10) | 4.30 (3.92–4.69) | 5.25 (4.78–5.72) |
| **2008** | 4.91 (4.61–5.22) | 4.41 (4.03–4.80) | 5.42 (4.94–5.89) |
| **2009** | 5.20 (4.89–5.50) | 4.28 (3.91–4.66) | 6.20 (5.70–6.70) |
| **2010** | 6.03 (5.70–6.36) | 5.28 (4.87–5.69) | 6.81 (6.29–7.34) |
| **2011** | 6.81 (6.46–7.16) | 6.08 (5.64–6.52) | 7.49 (6.94–8.03) |
| **2012** | 7.48 (7.12–7.84) | 6.62 (6.16–7.07) | 8.28 (7.72–8.84) |
| **2013** | 7.97 (7.60–8.34) | 6.97 (6.51–7.44) | 8.97 (8.39–9.55) |
| **2014** | 8.87 (8.48–9.25) | 7.71 (7.23–8.19) | 9.98 (9.37–10.59) |
| **2015** | 10.28 (9.87–10.68) | 8.43 (7.93–8.92) | 12.31 (11.65–12.97) |
| **2016** | 10.80 (10.38–11.21) | 9.00 (8.50–9.50) | 12.75 (12.09–13.42) |
| **2017** | 12.50 (12.06–12.94) | 10.66 (10.11–11.21) | 14.55 (13.85–15.26) |
| **2018** | 13.11 (12.66–13.55) | 10.75 (10.20–11.29) | 15.73 (15.00–16.45) |
| **2019** | 14.39 (13.92–14.85) | 11.85 (11.29–12.41) | 17.08 (16.33–17.83) |
| **2020** | 21.18 (20.63–21.74) | 17.39 (16.71–18.06) | 25.28 (24.39–26.18) |
| **2021** | 27.44 (26.81–28.09) | 22.22 (21.45–23.01) | 33.12 (32.10–34.17) |
| **2022** | 23.84 (23.26–24.43) | 19.92 (19.21–20.66) | 28.06 (27.13–29.01) |
| **2023** | 21.32 (20.77–21.87) | 17.30 (16.65–17.98) | 25.76 (24.88–26.67) |
| **2024** | 20.82 (20.29–21.36) | 17.09 (16.44–17.75) | 24.87 (24.02–25.75) |

**Supplementary Table 2.** Obesity and atrial fibrillation age-adjusted mortality rates per 1,000,000, stratified by race in the United States, 1999 to 2024.

|  | **Age-Adjusted Rate (95% CI)** | |
| --- | --- | --- |
| **Year** | **Black or African American AAMR (95% CI)** | **White AAMR (95% CI)** |
| **1999** | 2.58 (1.87–3.47) | 1.86 (1.65–2.08) |
| **2000** | 2.09 (1.45–2.93) | 2.36 (2.11–2.60) |
| **2001** | 2.88 (2.14–3.80) | 2.41 (2.17–2.65) |
| **2002** | 2.74 (2.03–3.62) | 2.77 (2.51–3.03) |
| **2003** | 2.72 (2.02–3.58) | 3.05 (2.78–3.32) |
| **2004** | 3.65 (2.83–4.63) | 3.29 (3.01–3.56) |
| **2005** | 3.72 (2.89–4.73) | 3.84 (3.55–4.14) |
| **2006** | 4.11 (3.24–5.13) | 4.20 (3.89–4.50) |
| **2007** | 4.80 (3.86–5.91) | 4.95 (4.62–5.28) |
| **2008** | 5.03 (4.04–6.02) | 5.06 (4.73–5.39) |
| **2009** | 5.04 (4.06–6.01) | 5.40 (5.06–5.74) |
| **2010** | 4.72 (3.80–5.63) | 6.36 (6.00–6.73) |
| **2011** | 4.81 (3.90–5.73) | 7.28 (6.89–7.67) |
| **2012** | 7.69 (6.52–8.87) | 7.78 (7.39–8.18) |
| **2013** | 6.47 (5.43–7.50) | 8.43 (8.02–8.84) |
| **2014** | 7.97 (6.83–9.11) | 9.32 (8.89–9.74) |
| **2015** | 8.23 (7.08–9.38) | 11.07 (10.61–11.53) |
| **2016** | 9.16 (7.97–10.34) | 11.42 (10.96–11.88) |
| **2017** | 10.66 (9.42–11.89) | 13.26 (12.77–13.76) |
| **2018** | 11.14 (9.86–12.43) | 14.05 (13.55–14.56) |
| **2019** | 11.62 (10.34–12.89) | 15.40 (14.88–15.93) |
| **2020** | 21.47 (19.75–23.19) | 22.12 (21.50–22.75) |
| **2021** | 25.86 (24.00–27.85) | 29.12 (28.40–29.85) |
| **2022** | 22.18 (20.47–24.01) | 25.52 (24.86–26.20) |
| **2023** | 20.41 (18.79–22.14) | 22.66 (22.04–23.29) |
| **2024** | 20.23 (18.65–21.92) | 22.20 (21.60–22.82) |

**Supplementary Table 3.** Obesity and atrial fibrillation age-adjusted mortality rates per 1,000,000, stratified by states in the United States, 1999 to 2020.

| **State** | **AAMR (95% CI)** |
| --- | --- |
| **Vermont** | 23.5 (20.6-26.4) |
| **Oklahoma** | 15.7 (14.7-16.8) |
| **Oregon** | 15.2 (14.2-16.2) |
| **Minnesota** | 15.1 (14.3-15.9) |
| **Wyoming** | 13.8 (11.3-16.3) |
| **Washington** | 13.5 (12.8-14.3) |
| **Wisconsin** | 12.9 (12.2-13.6) |
| **Iowa** | 12.5 (11.5-13.5) |
| **Colorado** | 11.4 (10.6-12.2) |
| **Idaho** | 11.2 (9.8-12.6) |
| **Alaska** | 10.6 (8.2-13.5) |
| **Rhode Island** | 10.1 (8.6-11.6) |
| **Nebraska** | 10.1 (8.9-11.2) |
| **South Dakota** | 9.9 (8.2-11.6) |
| **New Hampshire** | 9.9 (8.5-11.2) |
| **Indiana** | 9.8 (9.2-10.5) |
| **Montana** | 9.7 (8.2-11.2) |
| **North Dakota** | 9.6 (7.8-11.4) |
| **Ohio** | 9.4 (9.0-9.8) |
| **West Virginia** | 9.2 (8.2-10.2) |
| **South Carolina** | 8.7 (8.1-9.4) |
| **Pennsylvania** | 8.6 (8.2-8.9) |
| **Maryland** | 8.5 (7.9-9.1) |
| **Delaware** | 8.4 (7.0-9.9) |
| **Maine** | 8.4 (7.2-9.5) |
| **Tennessee** | 8.3 (7.7-8.8) |
| **Kentucky** | 8.2 (7.5-8.9) |
| **North Carolina** | 8.2 (7.7-8.6) |
| **California** | 8.0 (7.8-8.2) |
| **Kansas** | 7.5 (6.7-8.3) |
| **Texas** | 7.5 (7.2-7.8) |
| **New Jersey** | 7.2 (6.7-7.6) |
| **Missouri** | 6.9 (6.4-7.5) |
| **Hawaii** | 6.8 (5.7-7.9) |
| **Louisiana** | 6.5 (5.9-7.1) |
| **Mississippi** | 6.4 (5.6-7.1) |
| **Utah** | 6.2 (5.3-7.0) |
| **Illinois** | 6.1 (5.8-6.5) |
| **Michigan** | 6.1 (5.7-6.5) |
| **Arizona** | 5.9 (5.4-6.3) |
| **Florida** | 5.8 (5.5-6.0) |
| **Arkansas** | 5.6 (5.0-6.3) |
| **D.C.** | 5.4 (4.0-7.2) |
| **New Mexico** | 5.1 (4.3-5.9) |
| **Virginia** | 5.1 (4.7-5.5) |
| **New York** | 4.8 (4.6-5.1) |
| **Massachusetts** | 4.7 (4.2-5.1) |
| **Nevada** | 4.6 (4.0-5.3) |
| **Alabama** | 4.6 (4.1-5.1) |
| **Georgia** | 4.5 (4.2-4.9) |
| **Connecticut** | 4.1 (3.5-4.6) |

**Supplementary Table 4.** Obesity and atrial fibrillation age-adjusted mortality rates per 1,000,000, stratified by states in the United States, 2021 to 2024.

| **State** | **AAMR (95% CI)** |
| --- | --- |
| Oklahoma | 70.8 (66.2–75.7) |
| South Carolina | 62.1 (58.5–65.8) |
| Wyoming | 62.3 (51.7–74.8) |
| Wisconsin | 51.2 (48.2–54.5) |
| Vermont | 50.8 (42.5–60.7) |
| Minnesota | 43.6 (40.7–46.8) |
| Oregon | 45.3 (41.9–48.9) |
| Rhode Island | 46.8 (40.2–54.3) |
| Idaho | 38.5 (33.8–43.8) |
| Washington | 38.0 (35.6–40.5) |
| North Dakota | 36.8 (29.4–45.7) |
| Montana | 35.6 (29.8–42.3) |
| Nebraska | 35.8 (31.3–41.0) |
| Colorado | 34.4 (31.7–37.3) |
| Iowa | 39.4 (35.8–43.4) |
| Delaware | 54.7 (47.5–62.9) |
| South Dakota | 35.6 (29.0–43.4) |
| Kansas | 29.6 (26.2–33.3) |
| Kentucky | 28.0 (25.4–30.9) |
| Tennessee | 25.8 (23.8–28.0) |
| Pennsylvania | 25.6 (24.2–27.1) |
| Arkansas | 23.6 (20.6–26.8) |
| Georgia | 23.5 (21.8–25.2) |
| Utah | 23.9 (20.7–27.6) |
| North Carolina | 21.4 (19.9–23.0) |
| Arizona | 21.5 (19.7–23.4) |
| Louisiana | 24.0 (21.5–26.8) |
| Maryland | 24.0 (21.9–26.2) |
| Indiana | 23.2 (21.2–25.4) |
| Florida | 16.4 (15.6–17.3) |
| Michigan | 16.4 (15.0–17.8) |
| Virginia | 16.0 (14.5–17.5) |
| District of Columbia | 23.3 (16.7–31.9) |
| Illinois | 14.7 (13.6–16.0) |
| New York | 14.2 (13.3–15.2) |
| Hawaii | 12.2 (9.4–15.9) |
| Massachusetts | 10.9 (9.6–12.3) |
| Connecticut | 8.2 (6.7–10.0) |
| Alabama | 16.6 (14.7–18.7) |
| Alaska | 16.4 (11.3–23.7) |
| California | 19.0 (18.2–19.8) |
| Missouri | 19.9 (18.0–22.0) |
| Nevada | 18.6 (16.0–21.5) |
| New Hampshire | 23.9 (19.9–28.7) |
| New Jersey | 13.2 (12.0–14.6) |
| New Mexico | 13.2 (10.7–16.3) |
| Mississippi | 26.5 (23.3–30.1) |
| Maine | 24.5 (20.5–29.2) |

**Supplementary Table 5.** Obesity and atrial fibrillation age-adjusted mortality rates per 1,000,000, stratified by census region in the United States, 1999 to 2024.

|  | **Age-Adjusted Rate (95% CI)** | | | |
| --- | --- | --- | --- | --- |
| **Year** | **Midwest** | **Northeast** | **South** | **West** |
| **1999** | 2.5 (2.0-2.9) | 1.6 (1.2-2.0) | 1.6 (1.3-1.9) | 2.0 (1.6-2.6) |
| **2000** | 2.4 (2.0-2.9) | 2.2 (1.8-2.8) | 1.9 (1.6-2.3) | 2.7 (2.2-3.3) |
| **2001** | 2.8 (2.3-3.3) | 2.5 (2.0-3.1) | 1.9 (1.6-2.3) | 2.7 (2.1-3.2) |
| **2002** | 3.3 (2.7-3.8) | 1.9 (1.5-2.4) | 2.5 (2.1-2.8) | 3.3 (2.7-3.9) |
| **2003** | 3.2 (2.7-3.7) | 2.9 (2.4-3.5) | 2.6 (2.2-3.0) | 3.4 (2.8-4.0) |
| **2004** | 3.9 (3.3-4.4) | 3.1 (2.6-3.7) | 2.7 (2.3-3.1) | 3.6 (3.0-4.2) |
| **2005** | 4.1 (3.5-4.7) | 3.7 (3.1-4.3) | 3.3 (2.9-3.8) | 4.0 (3.4-4.6) |
| **2006** | 4.4 (3.8-5.0) | 3.7 (3.1-4.3) | 3.5 (3.1-4.0) | 5.0 (4.3-5.7) |
| **2007** | 5.6 (4.9-6.3) | 3.8 (3.2-4.4) | 4.4 (3.9-4.8) | 5.5 (4.8-6.2) |
| **2008** | 6.1 (5.4-6.8) | 4.4 (3.8-5.1) | 4.1 (3.7-4.6) | 5.5 (4.8-6.2) |
| **2009** | 5.8 (5.1-6.5) | 4.3 (3.6-4.9) | 4.8 (4.3-5.3) | 6.3 (5.5-7.0) |
| **2010** | 6.9 (6.1-7.6) | 6.0 (5.2-6.7) | 5.2 (4.7-5.7) | 6.5 (5.8-7.3) |
| **2011** | 7.8 (7.0-8.6) | 6.5 (5.7-7.3) | 5.9 (5.4-6.4) | 7.5 (6.8-8.3) |
| **2012** | 7.9 (7.1-8.6) | 7.8 (6.9-8.6) | 6.5 (5.9-7.0) | 8.5 (7.7-9.3) |
| **2013** | 9.3 (8.5-10.2) | 7.4 (6.6-8.2) | 7.0 (6.4-7.6) | 8.8 (8.0-9.6) |
| **2014** | 10.6 (9.7-11.5) | 7.5 (6.7-8.3) | 7.9 (7.3-8.5) | 9.8 (8.9-10.6) |
| **2015** | 12.3 (11.3-13.3) | 9.1 (8.2-10.0) | 8.5 (7.9-9.1) | 12.3 (11.4-13.3) |
| **2016** | 13.0 (12.1-14.0) | 8.8 (8.0-9.7) | 9.4 (8.8-10.0) | 12.7 (11.7-13.6) |
| **2017** | 15.6 (14.5-16.6) | 11.0 (10.0-11.9) | 10.5 (9.9-11.2) | 14.2 (13.2-15.1) |
| **2018** | 15.3 (14.3-16.3) | 10.2 (9.3-11.1) | 12.1 (11.4-12.8) | 15.2 (14.2-16.2) |
| **2019** | 17.6 (16.4-18.7) | 11.6 (10.6-12.6) | 13.4 (12.7-14.1) | 15.2 (14.2-16.2) |
| **2020** | 24.9 (23.6-26.2) | 17.0 (15.9-18.2) | 20.7 (19.8-21.5) | 21.7 (20.6-22.9) |
| **2021** | 19.97 (18.73–21.29) | 31.49 (30.02–33.02) | 27.58 (26.56–28.63) | 29.43 (28.07–30.86) |
| **2022** | 18.95 (17.76–20.22) | 26.01 (24.70–27.39) | 23.76 (22.82–24.72) | 25.76 (24.50–27.06) |
| **2023** | 16.05 (14.96–17.22) | 22.47 (21.25–23.74) | 22.24 (21.35–23.17) | 22.91 (21.74–24.13) |
| **2024** | 15.52 (14.46–16.65) | 23.54 (22.32–24.82) | 21.97 (21.10–22.88) | 20.56 (19.47–21.71 |

**Supplementary Table 6.** Obesity and atrial fibrillation age-adjusted mortality rates per 1,000,000, stratified by urbanization in the United States, 1999 to 2020.

|  | **Age-Adjusted Rate (95% CI)** | |
| --- | --- | --- |
| **Year** | **Metropolitan** | **Non-Metropolitan** |
| **1999** | 1.8 (1.5-2.0) | 2.5 (2.0-3.1) |
| **2000** | 2.2 (2.0-2.5) | 2.4 (1.9-2.9) |
| **2001** | 2.4 (2.1-2.6) | 2.6 (2.1-3.2) |
| **2002** | 2.5 (2.3-2.8) | 3.4 (2.8-4.1) |
| **2003** | 2.8 (2.5-3.0) | 4.0 (3.3-4.7) |
| **2004** | 3.1 (2.8-3.4) | 4.0 (3.3-4.6) |
| **2005** | 3.5 (3.2-3.7) | 4.9 (4.2-5.6) |
| **2006** | 3.9 (3.6-4.2) | 5.1 (4.3-5.8) |
| **2007** | 4.6 (4.3-5.0) | 5.5 (4.8-6.3) |
| **2008** | 4.6 (4.3-4.9) | 6.4 (5.6-7.2) |
| **2009** | 4.9 (4.5-5.2) | 6.9 (6.1-7.8) |
| **2010** | 5.7 (5.4-6.1) | 7.3 (6.4-8.2) |
| **2011** | 6.4 (6.1-6.8) | 8.5 (7.6-9.4) |
| **2012** | 7.3 (6.9-7.7) | 8.5 (7.5-9.4) |
| **2013** | 7.5 (7.1-7.9) | 10.0 (9.0-11.0) |
| **2014** | 8.4 (8.0-8.8) | 10.9 (9.9-12.0) |
| **2015** | 9.6 (9.2-10.0) | 13.7 (12.5-14.8) |
| **2016** | 10.3 (9.8-10.7) | 13.6 (12.5-14.7) |
| **2017** | 11.8 (11.3-12.3) | 16.1 (14.8-17.3) |
| **2018** | 12.4 (11.9-12.9) | 16.6 (15.3-17.8) |
| **2019** | 13.1 (12.6-13.6) | 20.7 (19.3-22.1) |
| **2020** | 20.0 (19.4-20.6) | 27.2 (25.6-28.8) |
