# Supplementary figures and images for: Trends in Atrial Fibrillation‐Related Mortality Among Adults With Obesity in the United States From 1999 to 2024
Source: Obes Sci Pract. 2026 Jun 10;12(3):e70160. doi: 10.1002/osp4.70160 (PMC13250834; doi:10.1002/osp4.70160)

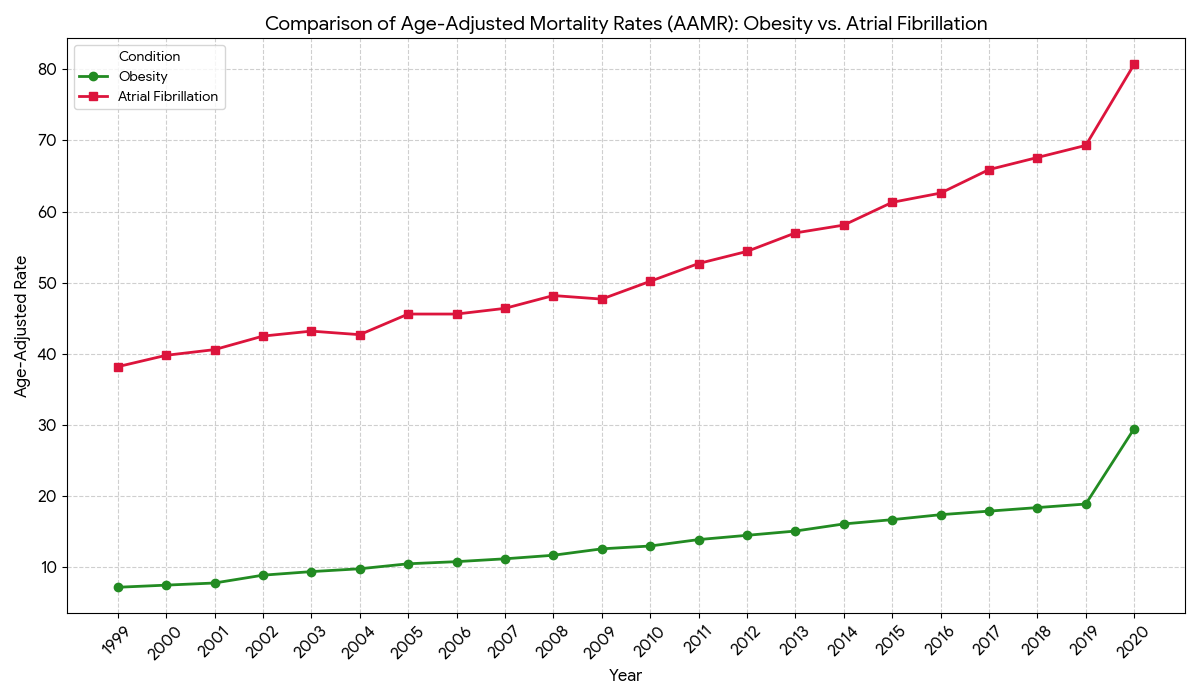

Supplement: Supplementary file 2 — Figure S1: Age‐adjusted mortality rates per 1,000,000 for obesity and atrial fibrillation as underlying or contributing causes of death, stratified by state in the United States, 1999–2020. [file OSP4-12-e70160-s005.png]

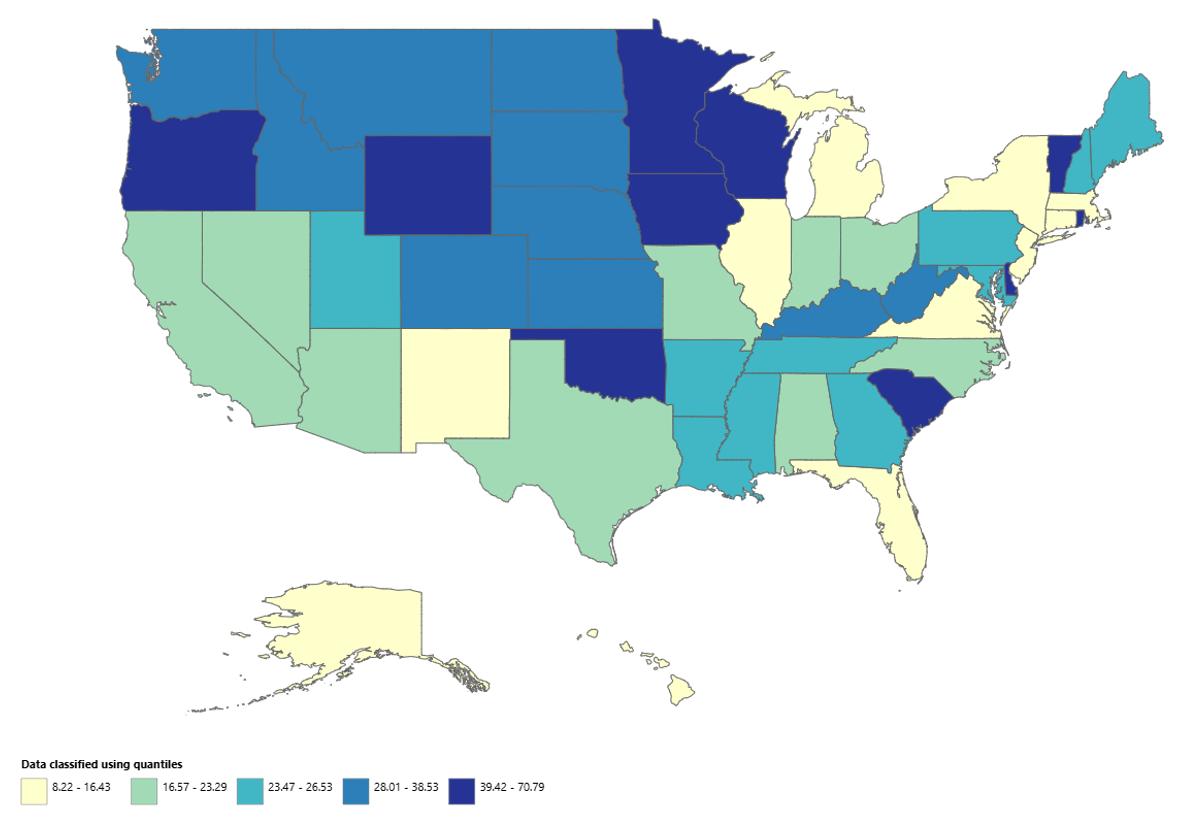

Supplement: Supplementary file 3 — Figure S2: Obesity and atrial fibrillation age‐adjusted mortality rates per 1,000,000, stratified by state in the United States, 2021 to 2024. [file OSP4-12-e70160-s001.png]

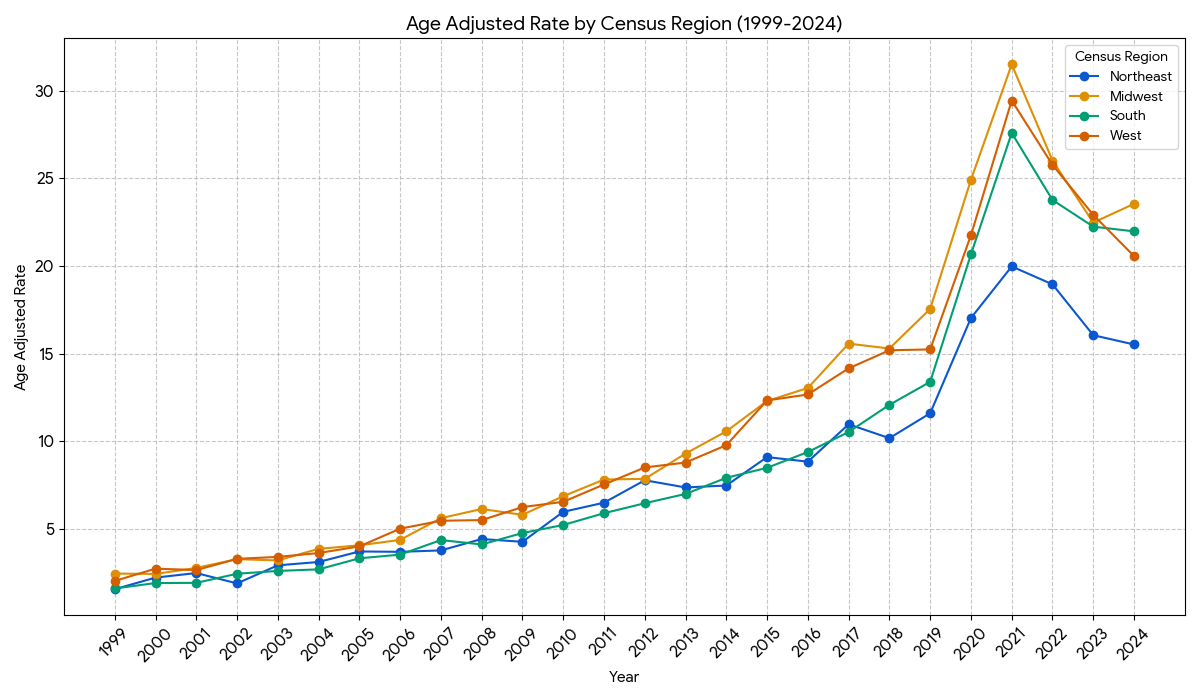

Supplement: Supplementary file 4 — Figure S3: Obesity and atrial fibrillation age‐adjusted mortality rates per 1,000,000, stratified by census region in the United States, 1999 to 2024. [file OSP4-12-e70160-s003.png]

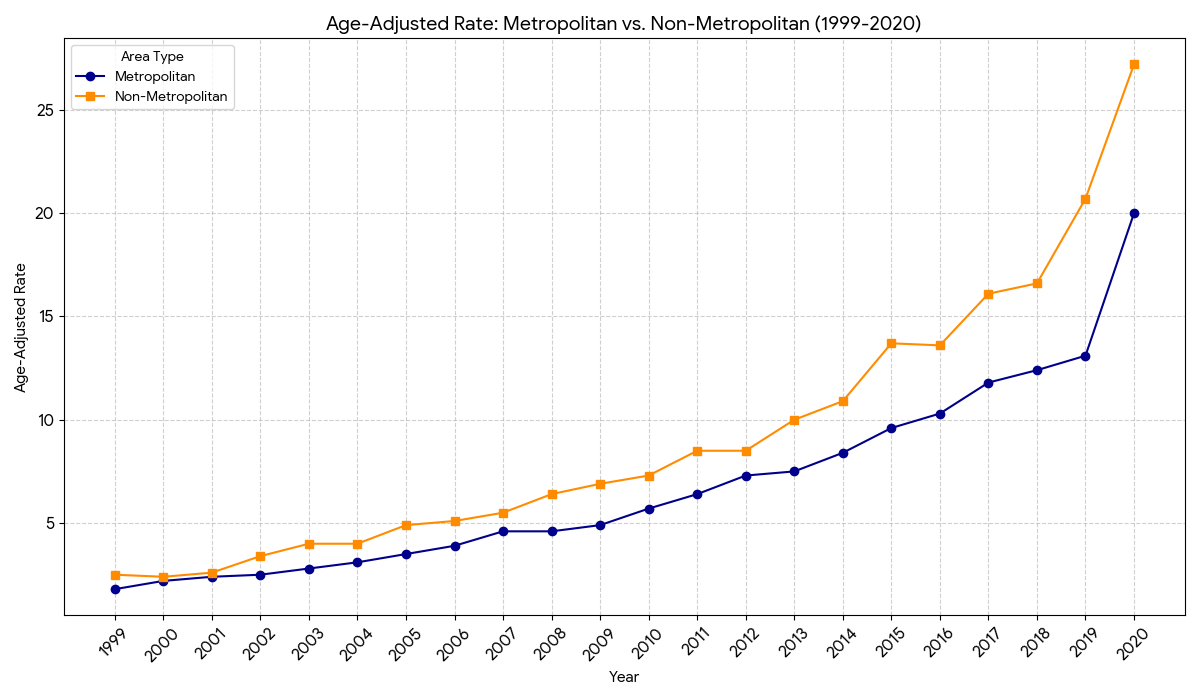

Supplement: Supplementary file 5 — Figure S4: Obesity and atrial fibrillation age‐adjusted mortality rates per 1,000,000, stratified by urbanization in the United States, 1999 to 2020. [file OSP4-12-e70160-s004.png]
